# STATegra EMS: an Experiment Management System for complex next-generation omics experiments

**DOI:** 10.1186/1752-0509-8-S2-S9

**Published:** 2014-03-13

**Authors:** Rafael Hernández-de-Diego, Noemi Boix-Chova, David Gómez-Cabrero, Jesper Tegner, Imad Abugessaisa, Ana Conesa

**Affiliations:** 1 Genomics of Gene Expression Lab, Centro de Investigación Príncipe Felipe, Valencia, Spain; 2 Computational Medicine. Karolinska Institute, Stockholm, Sweden; 3 Computational Genomics Program, Centro de Investigación Príncipe Felipe, Valencia, Spain

## Abstract

High-throughput sequencing assays are now routinely used to study different aspects of genome organization. As decreasing costs and widespread availability of sequencing enable more laboratories to use sequencing assays in their research projects, the number of samples and replicates in these experiments can quickly grow to several dozens of samples and thus require standardized annotation, storage and management of preprocessing steps. As a part of the STATegra project, we have developed an Experiment Management System (EMS) for high throughput omics data that supports different types of sequencing-based assays such as RNA-seq, ChIP-seq, Methyl-seq, etc, as well as proteomics and metabolomics data. The STATegra EMS provides metadata annotation of experimental design, samples and processing pipelines, as well as storage of different types of data files, from raw data to ready-to-use measurements. The system has been developed to provide research laboratories with a freely-available, integrated system that offers a simple and effective way for experiment annotation and tracking of analysis procedures.

## Background

The widespread availability of high-throughput sequencing techniques have importantly impacted genome research and reshaped the way we study genome function and structure. The rapidly decreasing costs of sequencing make these technologies affordable to small and medium size laboratories. Furthermore, the constant development of novel sequencing based assays, coined with the suffix *-seq*, expands the scope of cellular properties analyzable by high-throughput sequencing, with sequencing reads forming an underlying common data format. Today, virtually all nucleic acid omics methods traditionally based on microarrays have a -*seq *counterpart and many more have been made available recently. As a consequence, the possibility of running multiple sequencing-based experiments to measure different aspects of gene regulation and combining these with non-sequencing omics technologies such as proteomics and metabolomics has become practical [1-5]. For example, the ENCODE project combined ten major types of sequencing-based assays to unravel the complexity of genome architecture [6]. Many records can be found at the SRA archive that integrate multiple -*seq *technologies measured on the same samples and a PubMed search for NGS plus proteomics or metabolomics results in over hundred entries. Last but not least, one of the advantages of sequenced-based experiments is that they are equally applicable to the study of well-annotated model organisms as well as less-studied non-model organisms since little or no *a priori *genome knowledge is required.

Moreover, sequencing-based assays come with new challenges for data processing and storage. The memory size requirements of a medium sequencing experiments exceeds the capacity of current regular workstations. At the same time, the analysis steps to go from raw to processed data are more complex and resource intensive. As the number of datasets grows, the need to properly store and track the data and their associated metadata becomes more pressing. For example, a medium-sized RNA-seq experiments ranging between 4 to 20 samples of 20 million reads each can take up to 40 GB of raw data and generate multiple Quality Control and intermediate processing step files of up to 200 to 500 GB. Laboratory Information and Management Systems (LIMS) or Sample Management Systems (SMS) are bioinformatics tools that aid experimentalists to organize samples and experimental procedures in a controlled and annotated fashion. There are several commercial and free dedicated LIMS that have been developed specifically for genotyping labs where thousand of samples are processed by automated pipelines and procedures are tightly standardized [7-9]. One popular LIMS for genomics is BASE [10]. This software includes a highly structured system for metadata annotation and a flexible architecture for defining experiments and incorporating analysis modules. However, BASE is currently restricted to the annotation of microarray experiments.

Several laboratory information systems have been developed and implemented specifically for samples at sequencing facilities to manage the large volume of samples and data routinely handled by such services. Some of these have been made available to the scientific community or exported to other centers [11] published an extension of the Protein Information Management System (PIMS) for the Leeds University DNA sequencing facility designed to provide sample tracking both to users and operators. The system allows facility users to place orders and monitor the processing status of their samples while a different interface provides operators with a full control on the progression of the sequencing pipeline with automated connection to sequencing robots. The Leeds system supervises the whole procedure from sample submission to generation of fastq files but does not track the actual experimental characteristics of the sequenced samples or the post-processing of the raw data. Other solutions add to the tracking of sequencing samples analysis modules that execute some steps of the raw data processing such as Quality Control analysis or mapping to a reference genome. For example, the QUEST software [12] uses an experiment-resolved configuration file to store experiment metadata and execute predefined processing pipelines. Another example is NG6 [13] an integrated NGS storage and processing environment where workflows can be easily defined and adapted to different data input formats. NG6 can be used interactively to generate intermediate analysis statistics and downloadable end results. Similarly, Scholtalbers et al. recently published a LIMS for the Galaxy platform that keeps track of input sample quality and organize flow cells [14]. By working within the Galaxy system, associated fastq files are readily available for processing using the platform's analysis resources. Another interesting package is the MADMAX system that considers multiple omics experiments by incorporating modules for microrarrays, metabolomics and genome annotation [15]. MADMAX uses an Oracle relational database to store sample and raw data, and links to common bioinformatics tools such as Blast or Bioconductor installed on a computer cluster to facilitate data analysis.

We describe the STATegra Experiment Management System (EMS), which is an information system for storage and annotation of complex NGS and omics experiments. In contrast to other solutions that put the focus on management of thousands of samples for core sequencing facilities, the STATegra EMS has as primary goal the annotation of experiments designed and run at individual research laboratories. The system contains modules for the definition of omics experiments, samples and analysis workflows and it is able to incorporate data from different analytical platforms and sequencing services with great flexibility. The STATegra EMS supports currently mRNA-seq, ChIP-seq, DNase-seq, Methyl-seq, miRNA-seq, Proteomics and Metabolomics by default and can be easily adapted to support additional high-throughput experiments. The system uses free, open source software technologies, such as Java Servlets, the Sencha EXT JS framework, MySQL relational database system and the Apache Tomcat Servlet engine. The application can be downloaded from http://stategra.eu/stategraems.

## Methods

### STATegra EMS architecture

**Figure 1:**
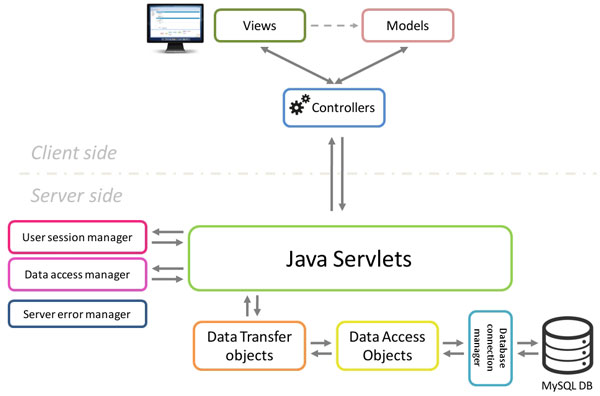
**Overview of the STATegra EMS architecture**.

The STATegra EMS was designed as a multiuser web application and is divided in two components: the SERVER SIDE application and the CLIENT SIDE web application (Figure 1).

The server side is the responsible for keeping the consistency of data and for controlling the access to the stored information, is built using Java Servlets and a MySQL relational database and is unique for all clients. Although primarily designed and tested on Linux servers, the server EMS code could easily be adapted to work over other architectures due to the cross-platform nature of Java. Additionally, the server code was implemented using the Data Access Object design pattern in conjunction with the Data Transfer Object pattern. This provides an abstraction layer for interaction with databases that acts as an intermediary between server application (servlets) and the MySQL database, making easier future extensions of the application code with new features or changes in the database model.

The STATegra EMS client side was developed as user-friendly and intuitive web application using Ext JS, a cross-browser JavaScript framework which provided powerful tools for building interactive web applications. The client side is based on the Model-View-Controller architecture pattern, which make easier to organize, maintain and extend large client applications. Communication between Client and Server side is handled by AJAX and HTTP GET and POST protocols using JavaScript Object Notation (JSON) for data exchange.

### User administration

The STATegra EMS is a system with user control. Users should be registered by the Administrator in the application before start working. As a general rule, the user creating a data element becomes the owner of this element and has exclusive rights for editing and deleting. However, any owner can grant access rights to other registered users of the system.

### Data specification

**Figure 2:**
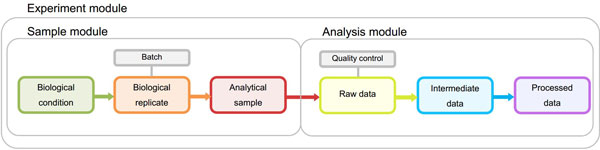
**Metadata Module structure in STATegra EMS**. The Sample module stores information of biological conditions, biological replicates and the associated analytical samples. The analysis module contains all analysis steps from raw to processed data. Both samples and analyses are associated to one or more experiments within the Experiment module.

The overall objective of the STATegra EMS is to serve as a logbook for high-throughput genomics projects performed at research labs by providing an easy-to-use tool for the annotation of experimental design, samples, measurements, and the analysis pipelines applied to the data. Experimental data and metadata are organized in the EMS around three major metadata modules (Figure 2): the *Experiment module *that records experimental design information and associated samples; the *Samples module *that collects all information on the used biomaterial; and the *Analysis module *that contains analysis pipelines and results. Both Sample and Analysis modules have been defined broadly to accommodate data from different type of omics experiments and still provide a common annotation framework. Commonly used standards in omics experimental data annotations were used when defining data specifications to facilitate EMS interoperability. In particular, we leveraged MIAPE [16] for proteomics analysis annotation, metabolomics guidelines proposed by [17] and [18] and MIAME [19] and MINSEQE [20] for sequencing experiments.

Sample and Analysis modules contain distinct *Information Units *(IUs), which are the basic elements of data input into the system and are connected by an experimental or analysis workflow. The Experiment Module is a wrapper of Samples and Analyses with one single data input form.

(i) Experiment module: The experiment is the central unit of information of the STATegra EMS. An Experiment is defined by some scientific goals and a given experimental design that addresses these goals. This design implies a number of biological samples and an array of omics measurements, which are assigned to the Experiment.

(ii) Sample module. This section hosts the information about biological conditions and their associated biological replicates and analytical samples. The IUs of this module are:

*Biological Condition*. These are defined by the experimental design and consist of a given biological material such as the organism, cell type, tissue, etc. and, when applicable, an experimental condition such as treatment, dose or time-point for time-series samples.

*Biological Replicate*. Each Biological Condition is assessed by using one or more biological replicates that may or may not correspond to the same experimental batch. The Biological Replicate stems directly from Biological Condition by adding a replicate number and, if applicable, a batch number.

*Experimental Batch*. Frequently, when an experiment is composed of a large number of samples, only some of them can be generated at the same time. These samples correspond to the same batch. Batch information is relevant to identify systematic sources of noise that might affect all samples within the batch.

*Analytical Sample*. Omics experiments analyze molecular components of biological samples using a given experimental protocol with the resulting analytical sample ready-to-be-measured by the high-throughput techniques. For example, a RNA-seq analytical sample is obtained after using a cytosolic mRNA extraction protocol. Similarly for metabolomics, different analytical samples can be obtained by applying multiple extraction protocols that target distinct metabolic compounds.

(iii) Analysis module. The Analysis module stores high-throughput molecular data obtained by the omics technologies and the data generated after processing of the primary raw data files. In contrast to the Sample module where only metadata is stored, the Analysis module also stores pointers to data files. The Analysis module consists of three data and one logical IUs:

*Raw Data*. These files contain the data as produced by the omics equipment. For example, fastq files in the case of sequencing experiments and NMR .raw files in the case of metabolomics experiments. The Raw data IU also contains detailed information of the experimental protocol applied to the analytical sample, i.e., the library preparation protocol followed in a RNA-seq experiment or the NMR analysis characteristics in the case of metabolomics.

*Intermediate Data*. This IU covers all processing steps from raw data to process data. Different omics experiment might require zero, one, or several intermediate steps. For example, in the case of RNA-seq, the mapping to a reference genome that produces a bam file constitutes an intermediate step. ChIP-seq will generally have two intermediate steps consisting of read mapping and peak calling.

*Processed Data*. The Processed data IU contains the final processing step that result in a data file containing the final signal values for the omics assay.

**Figure 3:**
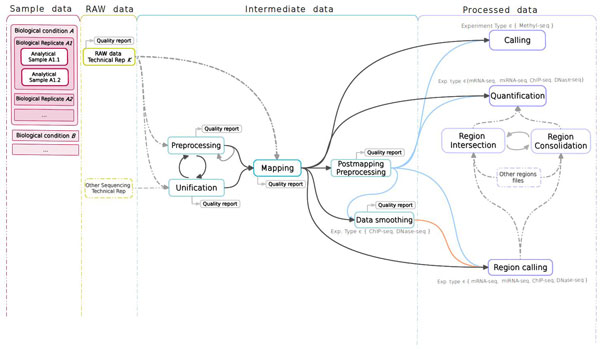
**STATegra EMS analysis workflow components**. The workflow is linked to an analytical sample object and consists of raw, intermediate and processed data IUs.

**Figure 4:**
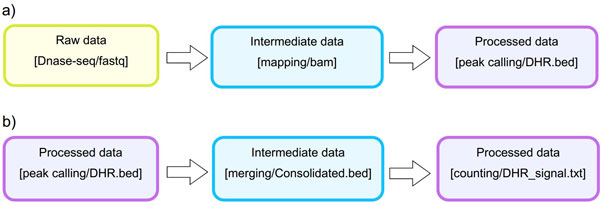
**Example of primary and secondary workflow for a DNase-seq analysis**. Primary workflow **(a) **involves calling DNase hypersensitivity regions (DHR) by applying a peak-calling algorithm to a BAM file of mapped reads whereas secondary workflow **(b) **involves merging of DHR.bed files from different samples to obtain a set of consolidated regions and then counting the number of reads of each sample in the consolidated region set to generate a per-sample signal value file.

*Analysis*. The STATegra EMS includes an additional IU, the *Analysis*, which is constructed by connecting some of the previous data IUs to define a data processing workflow. Figure 3 shows a generic representation of the workflow elements used in sequencing data analyses. An *Analysis *will start on a raw data file obtained from a particular analytical sample, continue through one or several intermediate data files covering different processing steps (such as trimming, mapping, filtering, merging, etc), and finalize in a processed data file that contain the signal values of the omics features. Alternatively, an *Analysis *can take as input a processed data file and apply additional processing steps to render a higher-level processed data. For example, in DNase-seq analysis, a primary workflow would be to call DNase hypersensitivity regions (DHR) by applying a peak-calling algorithm to a BAM file of mapped reads (Figure 4A); whereas a secondary *Analysis *could involve merging DHR.bed files from N different samples to obtain a set of consolidated regions and then counting the number of reads of each sample in the consolidated region set to generate a per-sample signal value file (Figure 4B).

**Figure 5:**
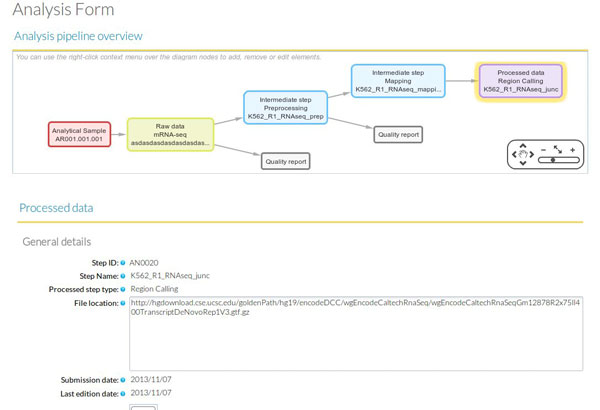
**Analysis module input window**.

In terms of data consistency, a unique Analysis ID is always associated to one Processed Data ID and describes the set of steps involved in obtaining that particular processed data. Moreover, an *Analysis *is always associated to one or more Experiments and, since the *Analysis *workflow can be traced back to raw data and its associated analytical samples, the *Analysis *provides the link between the Experiment and the Sample modules. By default, when a new *Analysis *is created, it will be assigned to the currently active Experiment. Figure 5 shows the data input window at the Analysis module. The central panel displays the input form for the different analysis steps, while at the bottom a graphical representation of the workflow allows easily monitoring the elements and structure of the *Analysis*.

## Results

### Use case

**Figure 6:**
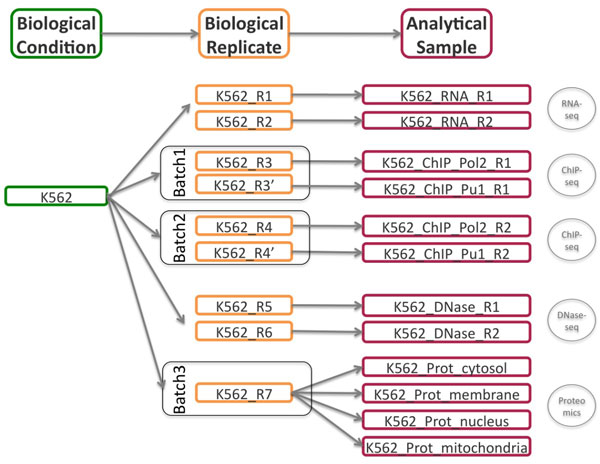
**Sample scheme for cell line K562 ENCODE user case data**. See main text for description.

To illustrate the usage and functionalities of the STATegra EMS, we consider the registration into the system of a subset of the ENCODE human dataset [21] comprising the four omics data types (mRNA-seq, ChIP-seq, DNase-seq and Proteomics) of two cell lines GM12878 and K562, with two biological replicates for most data types. ENCODE cell lines were regularly cultured in batches at data production labs to generate the samples for the different sequencing assays. In our example, we consider the utilization of one or several biological replicates from the same batch to obtain the analytical samples for ChIP-seq and proteomics experiments as depicted in Figure 6. An instance of the STATegra EMS with the ENCODE test data fully annotated into the system can be accessed at http://stategra.eu/stategraems_test.

**Figure 7:**
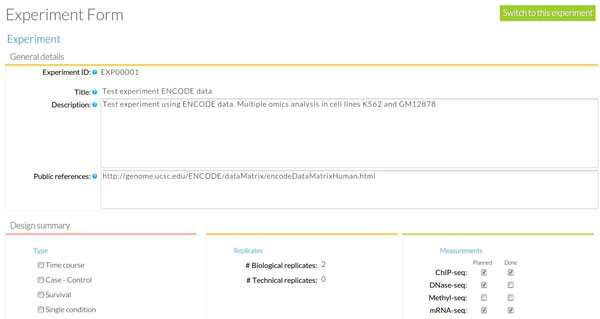
**Annotation details at Experiment module**.

At the Experiment Module a new experiment is created by the Experiment owner who assigns registered users to it. The Experiment has a unique ID within the system along with some basic information such as goal, description, type of experiment, experimental design and planned omics measurement types. Check boxes next to each planned measurement are available to monitor the progress of the experiment. These are automatically checked when a matching Analysis is uploaded and assigned to the Experiment. Figure 7 shows the Experiment annotation for our use case: a human-readable name (*ENCODE test*) and description (*STATegra EMS test experiment*) are given for the experiment, and the basic experimental details are indicated: *multiple conditions *experiment type, *two biological replicates *and three omics types, *mRNA-seq*, *ChIP-seq*, *DNase-seq *and *proteomics*.

**Figure 8:**
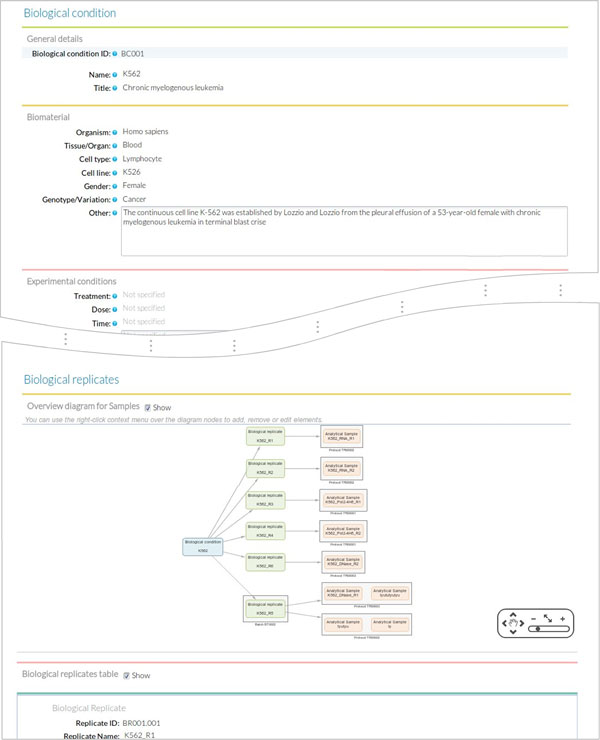
**Sample form**. The sample form provides fields to annotate biological condition details including data on the associated biological replicates and analytical samples.

Users then fill the Sample Module input form (Figure 8). The first of four sections of this form includes fields for *General *information on the sample such as *sample ID *(auto_generated), sample *name *(K562 or GM12878) and *title*, which is a more extended description of the sample (Chronic myelogenous leukemia/Lymphoblastoid). The second section of the form is used to describe the *Biomaterial*. For this use case, we entered the lymphocyte *cell type *from the blood *tissue *in human, with GM12878 as normal and K562 as cancer *variation*. The *Experimental condition *section is left blank in this example, since our cell lines did not receive any particular treatment. The next section records the associated *Biological Replicates*, where we can *add *items when more than one biological replicate is available for the same biological condition. For this user case, we created nine biological replicates to implement the scheme in Figure 6 and indicated the number for each replicate (i.e. #1), the corresponding *batch *when applicable, and its derived *analytical samples*. Analytical samples are characterized by an *extraction protocol *and a *name*. Multiple analytical replicates can be created for one biological replicate. For example, the RNA-seq data in our use case has one Analytical sample per each of the two biological replicates obtained with the Caltech long mRNA extraction protocol, while the proteomics data includes four analytical samples corresponding to each sub-cellular fraction (membrane fraction, cytosol, nucleus, mitochondria) (Figure 6).

Finally, information about the processing workflows is incorporated in the Analysis module. While the use case involves four omics types, we will only describe the RNA-seq workflow in detail, as the Chip-seq, DNase and Proteomics workflows are conceptually similar.

Within the active experiment, a new *Analysis *is selected, indicating mRNA-seq as the analysis type. Clicking on the "Annotate new step" adds each new analysis step. At the beginning of an Analysis "Raw Data" is the only option available, which opens the Raw Data form. At this point the user selects an existing Analytical Sample ID to start annotating the library preparation details and sequencing characteristics of a particular sample. In our example, we would choose the Analytical Sample #1 corresponding to Biological Replicate #1 of the K562 cell line. Additionally we can indicate the location of the raw data fastq file in our system. In principle the EMS does not stores any raw or processed data files, and instead provides pointers to the location of these files. Once the Raw Data form is completed and saved, a graphical representation of the analysis workflow is created on the lower screen window of the Analysis Module (Figure 5). This workflow will grow with the subsequent analysis steps. After the Raw Data step is created, additional intermediate steps can be added. In our user case, the next intermediate step to add is the mapping of the reads to the human reference genome, where the user would indicate which raw data file was used and other parameters such a reference genome annotation file or mapping algorithm such as GENCODE hg19 and TopHat respectively. Other subsequent intermediate steps can be envisioned such as trimming, removal of duplicate reads, etc. The analysis workflow ends with a Processed Data step with a form that requires annotation of a previous intermediate or raw data steps. For RNA-seq, one Processed Data step could for example correspond to the Cufflinks gtf file with FPKMs. A completed *Analysis *can be partially reused and modified to create additional workflows such as one leading to a different Processed Data step. For example, the first two steps of the previous analysis workflow can be imported to create a new workflow having a different Processed Data step recording a junction's bed file obtained by TopHat.

## Conclusion

As high-throughput sequencing costs decrease and new sequencing-based molecular assays become available more research laboratories incorporate the NGS technology as a tool to address their scientific goals. This is additionally promoted by the fact that high-throughput sequencing is also feasible in organisms with very little genome information. In a typical scenario, the researcher plans and outsources his/her experiments to sequencing facilities that might vary over time or according to the specific NGS assay required. When sequencing results arrive and accumulate, a necessity arises to properly store and organize large datasets and their associated processing pipelines. The STATegra EMS has been conceived to provide a management solution in these cases. The architecture of the system was designed having in mind the situation at research labs, where multiple experiments are run, samples might be replicated of reused in successive experiments, and one same biomaterial source could be used for different types of NGS assays. For this, the Sample Module arranges annotation into three Information Units: biological condition, biological replicate and analytical sample allowing one to many relationships, which creates the flexibility to define complex sampling settings without duplication of information. Similarly, the Analysis module divides metadata annotation into steps that can be reused to create alternative analysis workflows. Finally, by allowing samples and analyses to belong to different experiments, the STATegra EMS can accommodate possible connections between experiments.

This architecture is substantially different from other information management solutions created for NGS data that are oriented to sequencing facilities, such as the Galaxy LIMS [14] which handles requests from users to the service, or the NG6 [13] that controls the sequencing workflow at sequencing providers. In these cases the management system is adapted to the production pipeline at the sequencing center and applies a strong control on the facility wet-lab including library preparation and sequencer runs. This type of information is absent from the STATegra EMS, which may actually accept data from multiple sequencing providers. On the contrary the STATegra EMS records experimental information and sample metadata that might not be relevant at a production center. In conclusion, NGS LIMS and the STATegra EMS target different users and needs in the management of sequencing data. An open challenge is yet to optimize the integration of -seq data with clinical information similarly to what is done in clinical development centers [22,23].

The current STATegra EMS supports analysis workflows for five popular sequencing functional assays but can easily be extended to other *seq applications as processing step forms are generic for DNA and cDNA high-throughput sequencing. Additionally, the system supports annotation of omics experiments targeting non nucleic acid components, such as proteomics and metabolomics for which specific input forms have been incorporated. All together, the STATegra EMS provides an integrated system for annotation of complex high-throughput omics experiments at functional genomics research laboratories.

## Availability and requirements

The STATegra EMS application is distributed under GNU General Public License, Version 3 and can be obtained from http://stategra.eu/stategraems. The STATegra EMS was developed in JAVA and is therefore platform independent, but it has only been extensively tested for UNIX environments. The software MySQL server and Apache Tomcat. Installation instructions can be found at http://stategra.eu/stategraems_installation.

## Authors' contributions

RH conceived, implemented the STATegra EMS and helped drafting the manuscript. NBC helped implementing. IA, DGC and JT helped in conceiving the EMS. AC supervised the work and drafted the manuscript. All authors approved the final version of the manuscript.

## Competing interests

The authors declare no conflicts of interests.

## Declarations

The publication costs for this article were funded by the STATegra project, EU FP7 grant number 30600.

This article has been published as part of *BMC Systems Biology *Volume 8 Supplement 2, 2014: Selected articles from the High-Throughput Omics and Data Integration Workshop. The full contents of the supplement are available online at http://www.biomedcentral.com/bmcsystbiol/supplements/8/S2.
